# IFN*α*-Expressing Amniotic Fluid-Derived Mesenchymal Stem Cells Migrate to and Suppress HeLa Cell-Derived Tumors in a Mouse Model

**DOI:** 10.1155/2018/1241323

**Published:** 2018-04-02

**Authors:** Jun Zhou, Tian Liang, Dejun Wang, Liru Li, Yan Cheng, Qiuyan Guo, Guangmei Zhang

**Affiliations:** ^1^The First Affiliated Hospital of Harbin Medical University, Harbin, China; ^2^The Third Affiliated Hospital of Harbin Medical University, Harbin, China

## Abstract

**Background:**

Immunotherapy for cervical cancer with type I interferon (IFN) is limited because of the cytotoxicity that accompanies the high doses that are administered. In this study, we investigated the utilization of amniotic fluid-derived mesenchymal stem cells (AF-MSCs) as a means for delivering IFN*α* to local tumor sites for the suppression of cervical cancer in a mouse model using HeLa cell xenografts.

**Methods:**

The tumor tropism ability of AF-MSCs and AF-MSCs genetically modified to overexpress IFN*α* (IFN*α*-AF-MSCs) was examined through Transwell in vitro and through fluorescent images and immunohistochemistry in a mouse model. Tumor size and tumor apoptosis were observed to evaluate the efficacy of the targeting therapy. Mechanistically, tumor cell apoptosis was detected by cytometry and TUNEL, and oncogenic proteins c-Myc, p53, and Bcl-2 as well as microvessel density were detected by immunohistochemistry.

**Results:**

In this model, intravenously injected AF-MSCs selectively migrated to the tumor sites, participated in tumor construction, and promoted tumor growth. After being genetically modified to overexpress IFN*α*, the IFN*α*-AF-MSCs maintained their tumor tropism but could significantly suppress tumor growth. The restrictive efficacy of IFN*α*-AF-MSCs was associated with the suppression of angiogenesis, inhibition of tumor cell proliferation, and induction of apoptosis in tumor cells. Neither AF-MSCs nor IFN*α*-AF-MSCs trigger tumor formation.

**Conclusions:**

IFN*α*-AF-MSC-based therapy is feasible and shows potential for treating cervical cancer, suggesting that AF-MSCs may be promising vehicles for delivering targeted anticancer therapy.

## 1. Introduction

Cervical cancer, a malignant tumor of the female reproductive system, is a leading cause of mortality and a significant hazard to women's health worldwide. Although cervical cancer can be effectively treated by cystectomy in the early stages, the treatment of advanced cancer appears to be less efficient resulting in high morbidity rates. New molecular-based therapy has been developing rapidly [1]. Because human papillomavirus (HPV) infection is a known cause of cervical cancer [2], IFN*α*, an antiviral as well as antitumor cytokine, has been widely applied in clinical therapy of cervical cancer [3, 4]. IFN*α* is believed to not only be immunomodulatory but to also be able to suppress tumor growth by inducing apoptosis [5]. Nevertheless, due to the high dose of systemically administered IFN*α*, the side effect of cell toxicity is inevitable, and the short half-life of this protein also hinders it from reaching the desired concentration at tumor sites [6]. Currently, stem cells are being explored as a promising vehicle candidate for targeted therapy.

Mesenchymal stem cells (MSCs), a cell population that can be found in a variety of tissues, are capable of self-renewal and can be easily isolated and cultured because they can adhere to plastic [7, 8]. They are not only of low immunogenicity and oncogenicity, but they can also selectively migrate to tumor sites [2, 3, 9, 10]. There have been several reports on the use of MSCs for effective cytotherapy in diverse tumor models [11–15]. One study demonstrated that bone marrow (BM)-MSCs modified to express IFN*β* could also be specifically recruited to the tumor site and resulted in tumor suppression [12]. Additionally, Rachakatla et al. showed that in a model of breast carcinoma, MSCs isolated from the umbilical cord matrix (UCMS cells) exhibited similarly specific migration to the tumor and that the engineered cells secreting IFN*β* significantly reduced the tumor burden [16]. Although studies on the antitumor effects of MSCs from BM and other sources have been widely reported, few have focused on amniotic fluid-derived MSCs (AF-MSCs). AF-MSCs can be acquired through amniocentesis, which is less invasive and safer than bone marrow puncture. AF-MSCs have similar characteristics to human BM-MSCs but are less differentiated [17]. Due to their advantageous properties, including stable characteristics, nontumorigenicity, and low immunogenicity [18], AF-MSCs are emerging as a new candidate in regenerative medicine and anticancer therapy [17, 19]. Most existing studies on AF-MSCs are associated with their application in the field of regenerative medicine, especially in tissue repair in acute injury models [20–23]. Importantly, these studies take advantage of the innate ability of MSCs to migrate to inflammatory signaling sites. Therefore, researchers have deduced that AF-MSCs should be able to engraft to tumor sites, regardless of tissue origin, as they do in injury models, and serve as delivery vehicles for antitumor molecules [3, 13, 24, 25].

Based on the aforementioned studies of MSCs in antitumor applications, in this study, we performed investigations of the ability of AF-MSCs to migrate to cervical cancer cells in vitro and in vivo. In addition, we explored the efficacy of AF-MSCs, especially those engineered to express IFN*α*, in the treatment of cervical cancer.

## 2. Methods

### 2.1. Isolation and Characterization of AF-MSCs

Human AF-MSCs were isolated from the amniotic fluid in the second trimester of gestation. All clinical investigations were conducted according to the principles expressed in the Helsinki Declaration. The amniotic fluid samples (5–15 ml) were aspirated via amniocentesis under ultrasonographic control with informed consent. Each sample was centrifuged at 1000 rpm for 10 min, and the cell pellet was resuspended in *α*-Modified Eagle's Medium (*α*-MEM) (Hyclone, USA) containing L-glutamine supplemented with 15% fetal bovine serum (FBS, Gibco, USA) and a 1% penicillin-streptomycin mixture in 6-well plates and incubated at 37°C in a 5% humidified CO_2_ chamber. After 7 days, nonadherent cells were removed and the medium was discarded. The adherent cells were cultured in fresh medium until clones appeared. Subsequently, the medium was changed every 3 days, and the adherent cells were trypsinized and subcultured whenever they reached 80%–90% confluence.

To identify these cells as AF-MSCs, several cell surface markers specific for this type of stem cell (CD90, CD105, CD73, HLA-ABC, CD34, CD14, CD45, and HLA-DR) were assessed by flow cytometry. Furthermore, the capacity of AF-MSCs to differentiate into osteocytes was evaluated. AF-MSCs were seeded at 1 × 10^5^/cm^2^ in 6-well plates and cultured in an osteogenic medium (*α*-MEM supplemented with 10% FBS, 0.1 mmol/l dexamethasone, 10 mmol/l glycerol phosphate, and 50 mmol/l ascorbate). The medium was changed every 3 days, and the cells were stained with Alizarin red on day 21 to assess the presence of calcifying nodules.

### 2.2. HeLa Cells

HeLa cells, originally from a human cervical cancer, were a gift from Dr. Zheng (Department of Gynecology, First Affiliated Hospital of Harbin Medical University). The cells were cultured in 1640 medium (Hyclone, USA) supplemented with 10% FBS and a 1% penicillin-streptomycin mixture.

### 2.3. Preparation of IFN*α*-Overexpressing AF-MSCs

IFN*α* cDNA was reverse-transcribed and amplified from mRNA extracted from the peripheral blood mononuclear cells (PBMCs) obtained from Chinese volunteers. The HIV-1-based lentiviral transfer plasmid, pTY-CMV-eGFP, contains the enhanced green fluorescent protein (eGFP) reporter gene that is driven by the CMV promoter and was provided by Dr. C. Li (Southern Medical University, China). To express IFN*α* using this lentiviral vector, the eGFP gene was replaced with IFN*α* cDNA, and the resultant transfer plasmid was named pTY-CMV-IFN*α*. The transfer plasmid, pTY-CMV-IFN*α*, and the packaging plasmids, psPAX2 and pMD2.G (Addgene, USA), were used to transfect 293T cells with liposome 2000 (Invitrogen, USA) to produce VSV-G pseudotyped, IFN*α*-producing lentivirus. The transfected cells were incubated for 48 h to allow the production of recombinant virus. The recombinant viruses were harvested, titrated, and then used to infect AF-MSCs at a multiplicity of infection (moi) of 0.75 pg/ml (defined as the activity of viral reverse transcriptase) for 48 h. The retrovirus-infected, IFN*α*-producing cells (IFN*α*-AF-MSCs) were selected due to their resistance to puromycin. For further identification, IFN*α*-AF-MSCs were also subjected to stem cell surface marker test (CD90, CD105, CD73, HLA-ABC, CD34, CD14, CD45, and HLA-DR) by flow cytometry. The levels of IFN*α* expressed by IFN*α*-AF-MSCs and MSCs were detected by ELISA (Pbl Biomedical Labs, USA).

### 2.4. AF-MSCs Labeled with CM-DIL

To evaluate the tropism of MSCs for cancer cells in vivo, AF-MSCs and IFN*α*-AF-MSCs were labeled with a fluorescent cell surface marker, CM-Dil (Invitrogen), by incubation with a working solution of 5 *μ*g/ml CM-Dil for 5 min at room temperature, followed by a 20-minute incubation at 4°C. After labeling, the cells were washed with PBS and cultivated in fresh medium prior to injection into mice, which occurred within 24 h.

### 2.5. In Vitro Transwell Migration Assay

To determine the tropism of AF-MSCs and IFN*α*-AF-MSCs for HeLa cells in vitro, the Transwell migration assay was performed as previously described [13]. HeLa cells were cultured for 24 h in 1640 medium containing 10% FBS; after being trypsinized, HeLa cells were resuspended and plated at 1 × 10^6^/2 ml in the lower wells of Transwell chambers and allowed to adhere for 24 h. After being cultured in *α*-MEM (0.5% FBS) for 24 h, AF-MSCs and IFN*α*-AF-MSCs were plated in the upper well of chambers with 8 *μ*m pores (Corning Costar, USA) at a density of 1 × 10^5^/800 *μ*l. HSF, cultured in DMEM + 10% FBS previously, were used as control. These chambers were incubated at 37°C for 24 h to allow the MSCs to migrate. Then, the cells that had not migrated were removed from the upper chamber with a wet cotton swab, and the cells that had migrated were fixed and stained with trypan blue. Images of the stained cells that had migrated to the bottom of the Transwell chamber through the upper membrane were obtained using a Leica DMI4000B inverted phase-contrast microscope (Leica, Germany). The number of cells in 4 high-power fields (×200) per membrane was counted manually.

### 2.6. Analysis of Apoptotic HeLa Cells in the Presence of IFN*α*-AF-MSCs In Vitro

IFN*α* produced by IFN*α*-AF-MSCs was measured by ELISA in CM from P1-P5 IFN*α*-AF-MSCs. The CM was collected 48 hours post transduction from three random wells.

MSCs and IFN*α*-AF-MSCs from passages 5, 10, 15, and 20 were harvested and resuspended in a-MEM containing 10% (vol/vol) FBS at a concentration of 1 × 104 cells/ml, plated into 24-well plates at 5000 cells (500 *μ*l) per well, and incubated 24 hours in a humidified 37°C incubator under 5% CO_2_ to allow the cells to adhere to the plates. The cells from three random wells were counted for 8 days, and the mean values were used to plot cells.

The AF-MSCs and IFN*α*-AF-MSCs were cocultured with HeLa cells to investigate the cytopathic effect of MSCs on the cervical cancer cells. HeLa cells were plated on the lower well of 12-well Transwell plates at 5 × 10^5^ cells/ml in 1640 (10% FBS), while the MSCs were plated on the upper wells (0.4 *μ*m porous inserts) in *α*-MEM (10% FBS), and then cultured for 24 h. Then, the upper wells with AF-MSCs or IFN*α*-AF-MSCs were inserted into the bottom well containing HeLa cells, and the two layers of cells were cocultured in Transwell chambers for 72 h. HeLa cells cultured without MSCs were used as the negative control. HeLa cells were then trypsinized and stained with a FITC-labeled Annexin V Apoptosis Detection Kit II (BD, USA) according to the manufacturer's instructions before performing flow cytometry using a BD FACS Aria flow cytometer.

### 2.7. Animal Subjects

Female nude mice (Balb/c nude) were purchased from the Shanghai Slac Laboratory Animal Co. Ltd., and they were housed in the Animal Center of Harbin Veterinary Research Institute under specific pathogen free (SPF) conditions. The mice were held for 5 days after arrival to allow them to acclimatize before the experiments were carried out. All animal and human studies were performed in compliance with the US Department of Health and Human Services Guide for the Care and Use of Laboratory Animals and were approved by the Scientific Research Office of the First Affiliated Hospital of Harbin Medical University.

### 2.8. In Vivo Migration Assay

The eGFP-HeLa cells were injected subcutaneously at a concentration of 1 × 10^7^/ml into the backs of 6-week-old female Balb/c nude mice (day 0). Ten days later, when tumors were palpable, mice were given three doses of 5 × 10^6^/ml CM-Dil-dyed AF-MSCs (*n* = 3) or IFN*α*-AF-MSCs (*n* = 3) every 5 days and were sacrificed 4 days after the last injection of MSCs. For the tracking of fluorescent signals, tumors and organs (liver, lung, spleen, and kidney) were collected and made into cryosections and paraffin sections. The fluorescent images in cryosections were obtained via laser confocal microscopy (Leica, Germany).

Another set of mice (*n* = 12) was given AF-MSC intravenously (i.v.), and three mice from each group were sacrificed on day 1, day 3, day 7, and day 13. Tumors were collected to analyse the distribution of AF-MSCs in tumors with time. Immunohistochemistry (IHC) with an antihuman CD90 antibody was performed to track the MSCs in paraffin sections.

### 2.9. Tumor Analysis

For the establishment of tumors, 200 *μ*l of HeLa cell suspension (suspended in saline) was administered subcutaneously at a concentration of 1 × 10^7^/ml into the backs of 6-week-old female Balb/c nude mice. After 10 days, when the tumors were palpable, 1 × 10^6^ AF-MSCs (*n* = 12) or IFN*α*-AF-MSCs (*n* = 15) were administered i.v. into the tail vein at a volume of 200 *μ*l. The MSC injections were performed three times at 7-day intervals. Tumor-bearing mice administered with saline were designated as controls (*n* = 10). The tumors in all living mice were measured by calipers throughout the observation period.

One week after the last injection, randomly selected mice in each group (*n* = 3 for the control group and *n* = 5 for the AF-MSC and IFN*α*-AF-MSC groups) were sacrificed, and the tumors were collected. Secreted IFN*α* from IFN*α*-AF-MSC in tumors sites and other organs was tested by immunohistochemistry. To explore the degree of apoptosis in vivo, TUNEL staining was performed using the in situ Cell Death Detection Kit POD (Roche, USA) according to the manufacturer's instructions. TUNEL-positive cells were counted in 10 randomly selected fields at ×400 magnification in each tumor. Furthermore, IHC studies for the oncogenic proteins c-Myc, p53, and Bcl-2 were used to determine their expression. The expression levels of these apoptotic proteins were presented as the mean density by Image-Pro Plus (IPP 6.0).

In addition, microvessel density (MVD) was also evaluated in these tumors. MVD was assessed by staining with an antibody against the CD34 antigen (Dako, Denmark), and positivity was determined by light microscopy using the counting method introduced by Weidner et al. [26]. Briefly, the areas in the slides containing the maximum number of micro-blood vessels were chosen at low-power fields; individual microvessels were counted at ×200 magnification. Both isolated immunoreactive endothelial cells and luminal microvascular structures were considered as countable vessels.

### 2.10. Immunohistochemistry

The samples were fixed in 10% paraformaldehyde, dehydrated t, embedded in paraffin, and sectioned at 4 *μ*m. The sections were deparaffinized in dimethyl benzene for 30 min, 100% alcohol for 2 min, hydrogen peroxide for 10 min, and alcohol for 1 min each and washed. The slides were immersed in the corresponding retrieval solution and heated. After cooling, the sections were stained with the corresponding primary antibodies for 1 h at room temperature (anti-human CD90 (Bioworld, USA, 1 : 200), anti-human IFN*α* (Bioss, China, 1 : 300), anti-c-Myc (Ebioscience, China, 1 : 200), anti-P53 (Ebioscience, 1 : 300), anti-Bcl-2 (Abcam, Hong Kong, 1 : 200), and anti-CD34 (Dako, Denmark, 1 : 250)), rinsed in running tap water, stained with secondary antibody-1 (GBI, USA) for 20 min and secondary antibody-2 for 30 min, washed and developed with DAB, and counterstained with hematoxylin, hydrochloric acid, and ammonium hydroxide.

### 2.11. Evaluation of AF-MSC Tumorigenicity

Female Balb/c nude mice that were 4 weeks old were randomly assigned to experimental groups. AF-MSCs isolated from one single amniotic fluid sample, which presented great proliferation capacity at passages 13 to 15, were used for injection. IFN*α*-AF-MSCs and AF-MSCs were evaluated for their tumorigenicity. The mice were transplanted subcutaneously with 1 × 10^7^ AF-MSCs (*n* = 4) or IFN*α*-AF-MSCs (*n* = 4) suspended in 200 *μ*l of saline on the right side of their backs while the same amount of saline alone was administered on the other side as the control. All the mice were sacrificed 50 days after transplantation. The subcutaneous tissue of the injection sites, lung, kidney, liver, and spleen, was collected and fixed in 10% paraformaldehyde. HE staining was performed for the pathological diagnosis.

### 2.12. Statistical Analysis

Differences between groups were evaluated using the parametric or nonparametric Student's *t*-test or one-way analysis of variance. All reported *P* values were considered statistically significant at <0.05.

## 3. Results

### 3.1. AF-MSCs Were Isolated and Prepared for IFN*α* Overexpression

Human AF-MSCs were isolated and cultured as described in Materials and Methods section, and the first clone of adherent cells appeared 7 days after the initial plating. The cells appeared round, spindle shaped, or polygonal in the primary culture and gradually changed to a typical fibroblast-like spindle shaped with increasing passages. The cells were observed in a swirling or radial arrangement after being cultivated for 14 days, which was similar to the morphology of MSCs reported from other sources [7, 27]. To define the MSCs, specific markers of this stem cell were examined by flow cytometry. These cells were positive for CD90, CD105, CD73, and HLA-ABC and negative for CD34, CD14, CD45, and HLA-DR (Figure S1a). In addition, these cells differentiated into chondrogenic lineages, as previously reported [28] (Figure S1b). These results indicated that AF-MSCs were successfully isolated and cultivated. To develop AF-MSCs that consistently overexpressed INF*α*, these cells were infected with an HIV-1-based pseudovirus that contained the human INF*α* cDNA. Approximately 11,000 pg/ml IFN*α* was produced by 1 × 10^6^ IFN-MSCs 24 hours postinfection (hpi), as determined by assaying the culture medium using an IFN*α* detection ELISA kit. What is more, the stem cell marker (CD90, CD105, CD73, HLA-ABC, CD34, CD14, CD45, and HLA-DR) expression of IFN*α*-AF-MSCs MSC-alpha is actually consistent with the MSC null ones, which proved the cell stability. These IFN*α*-producing MSCs (IFN*α*-AF-MSCs) were used for the subsequent in vitro and mouse experiments.

### 3.2. AF-MSCs Migrated toward HeLa Cells In Vitro

**Figure 1:**
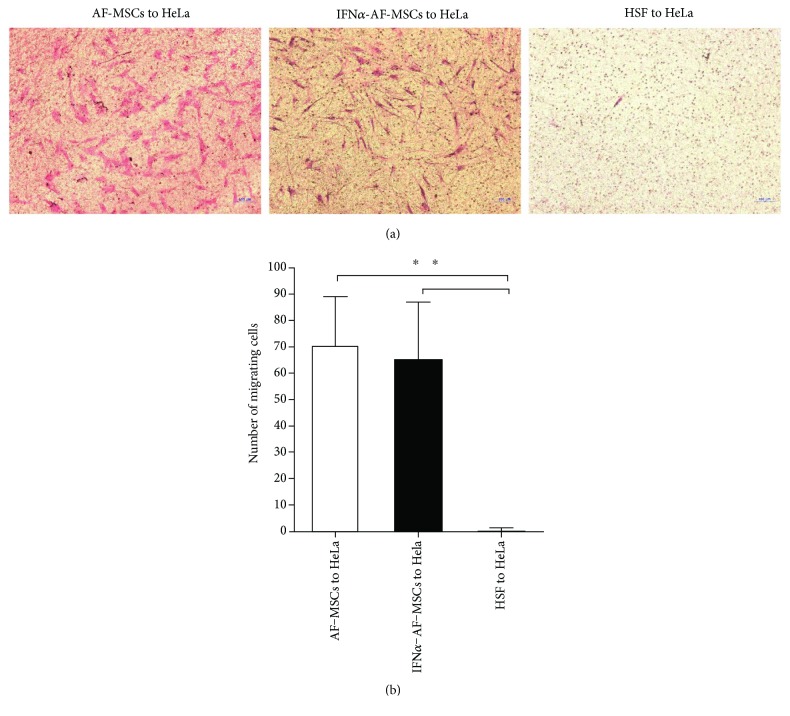
Migratory capacity of AF-MSCs and IFN*α*-AF-MSCs toward cultivated HeLa cells. (a) Images of the AF-MSCs and IFN*α*-AF-MSCs that migrated toward HeLa cells, as determined by the Transwell migration assay. HeLa cells were precultured in lower wells of the Transwell chambers for 24 h. AF-MSCs, IFN*α*-AF-MSCs, and HSF were then plated in the upper well of the chambers. 24 h later, stem cells that had migrated to the membrane were fixed and stained with trypan blue. The cells were counted by microscopy. Bar, 100 *μ*m. (b) Diagram of the migration capacity of AF-MSCs and IFN*α*-AF-MSCs. The cells of 4 high-power fields (×200) per membrane were counted manually. The data represent three independent experiments. Error bars, standard deviation, ^∗∗^ *p* < 0.01.

The tropism of AF-MSCs toward cancer cells was evaluated by examining the migration of AF-MSCs to HeLa cells, a cervical cancer cell line, using an in vitro Transwell system. As demonstrated in Figure 1, these stem cells exhibited significantly higher tropism to HeLa cells compared with HSF cells' migration toward HeLa (140-fold increase, *P* < 0.01). Similarly, the MSCs infected with IFN*α*-expressing lentivirus (IFN*α*-AF-MSCs) showed a great tropism to the HeLa cells. No significant differences in the migration of AF-MSCs and IFN*α*- AF-MSCs were observed.

### 3.3. Transplanted AF-MSCs Migrated toward Tumor Sites In Vivo

**Figure 2:**
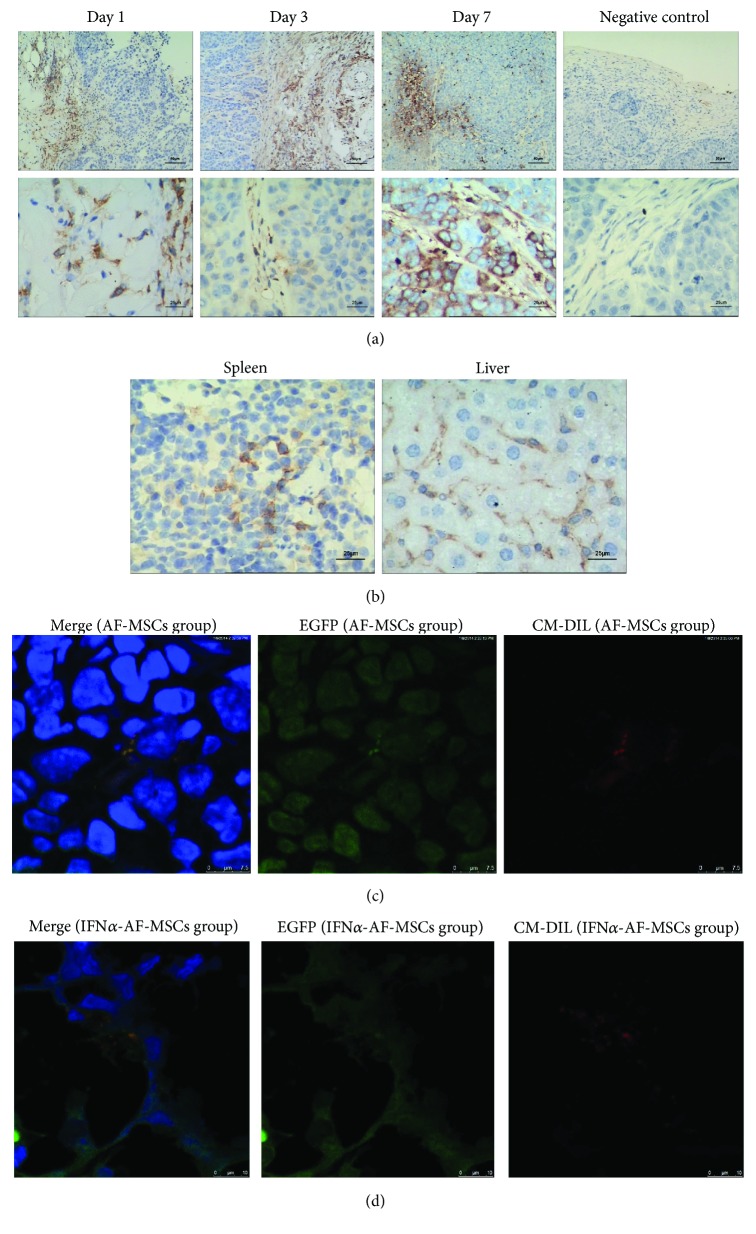
Tropism of administered AF-MSCs for tumors in a mouse model. (a) Immunohistochemistry (IHC) showing CD90 expression in AF-MSCs. The tumors were collected and examined at day 1, day 3, and day 7 after the last injection. Untreated tumor stained for CD90 is used as a negative control. Bars, 25 or 50 *μ*m. (b) IHC of AF-MSCs in the liver and spleen. Very few MSCs were observed. Bar, 25 *μ*m. (c, d) Observation of CM-Dil-labeled AF-MSCs and IFN*α*-AF-MSCs (in red) in the eGFP-HeLa (in green) tumors by fluorescence microscopy. Bars, 10, 25, or 50 *μ*m. HeLa cells that expressed eGFP (eGFP-HeLa) were injected subcutaneously into the backs of nude mice (*n* = 3) at 1 × 10^7^/ml/mouse. AF-MSCs and IFN*α*-AF-MSCs were labeled with the red fluorescent dye CM-Dil, and 10 days following the initial inoculation of HeLa cells, 5 × 10^6^/ml of either MSCs, or IFN*α*-AF-MSCs were injected into the tail vein of the tumor-bearing mice every 5 days. IHC and fluorescence microscopy were used to observe the infiltration of AF-MSCs.

Immunohistochemistry result of CD90 tracking (Figure 2(a)) showed that on day 1 after the administration of AF-MSCs, the AF-MSCs were mostly distributed throughout the periphery of the tumors and could hardly be found in the deep tumor regions. On day 3, some CD-90-positive cells began to appear inside the tumor, especially in the stromal regions consisting of nonmalignant tumor cells. On day 7 and day 13, more AF-MSCs were found to have infiltrated the tumor. Furthermore, besides the periphery and stromal region, many AF-MSCs were observed around areas of necrosis. A few AF-MSCs were identified in the spleen and perivessel parts of the liver throughout the study. No CD90-positive cells were found in negative controls. Cryosections showed that there were red fluorescence in edges and inside of tumor mass, AF-MSCs (Figure 2(c)) and IFN*α*-AF-MSCs (Figure 2(d)) were mostly observed in the connective tissue regions of the tumors, there was also a little red fluorescence in the spleen and liver, but no fluorescent signal can be seen in the kidney or lung tissues (Figure 2(b)).

### 3.4. IFN*α*-AF-MSCs Induced Apoptosis of Cocultured HeLa Cells

**Figure 3:**
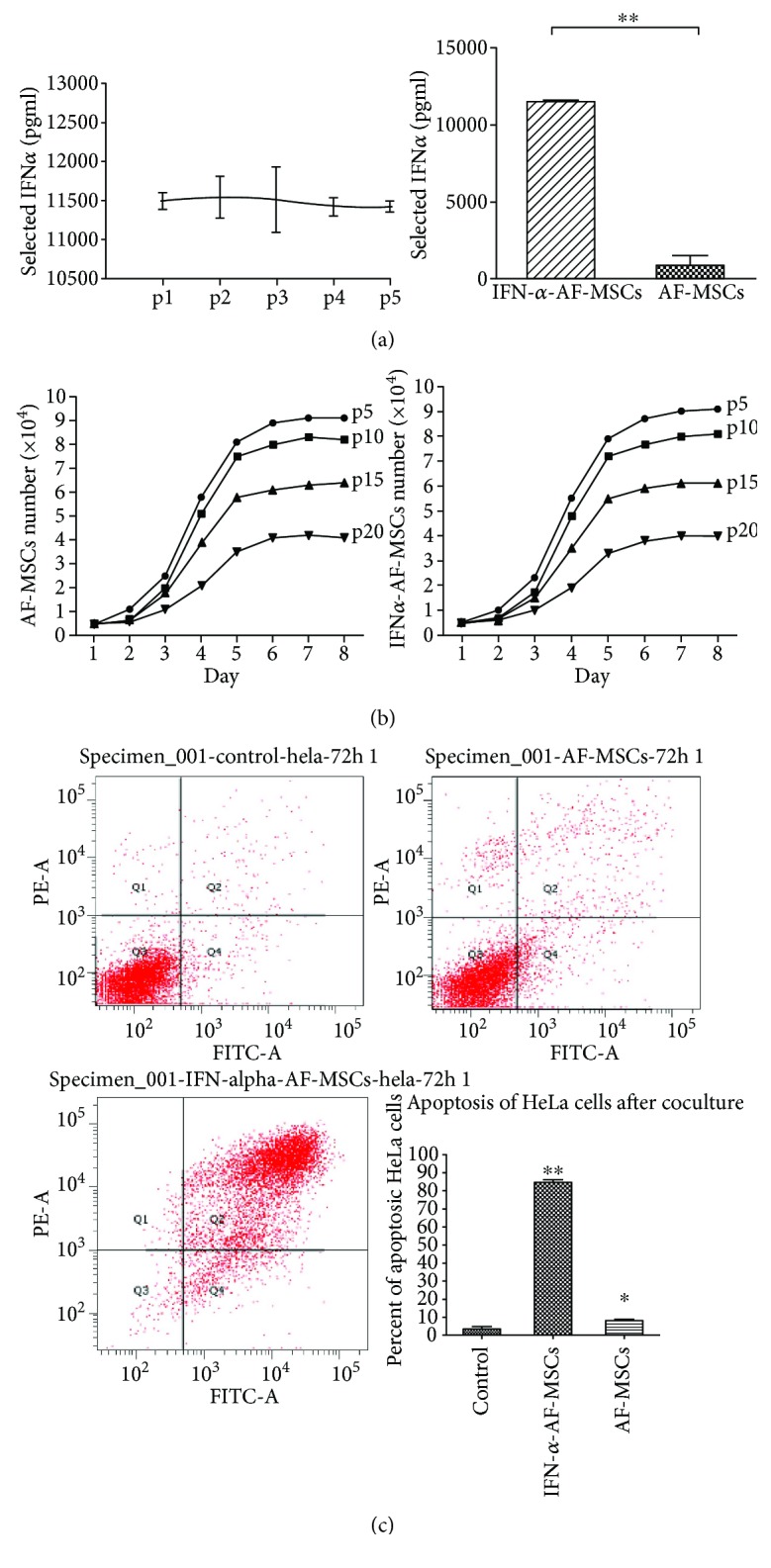
Induction of apoptosis of HeLa cells by IFN*α*-AF-MSCs in vitro. (a) Concentration of secreted IFN*α* in CM of IFN*α*-AF-MSCs of consecutive passage. IFN-*α* produced by IFN*α*-AF-MSCs and AF-MSCs was measured by ELISA in CM from P1-P5. The CM was collected 48 hours post transduction from three random wells. (b) Biological activity of the secreted IFN*α* on IFN*α*-AF-MSC proliferation. MSCs and IFN*α*-AF-MSCs from passages 5, 10, 15, and 20 were plated into 24-well plates at 5000 cells (500 *μ*l) per well, to allow the cells to adhere to the plates. The cells from three random wells were counted for 8 days, and the mean values were used to assess cell proliferation. (c) Induction of apoptosis of HeLa cells for coculture with IFN*α*-AF-MSCs. AF-MSCs or IFN*α*-AF-MSCs were cocultured with HeLa cells in 12-well Transwell plates for 72 h. HeLa cells cultured alone were the negative control. After coculture, the apoptotic populations of HeLa cells were evaluated via FITC-labeled annexin V/PI staining flow cytometry. Apoptotic populations, including the early (annexin V+/PI−) and late (annexin V+/PI+) apoptotic cells, were assessed in the HeLa cells cocultured with AF-MSCs and IFN*α*-AF-MSCs. The data represent three independent experiments. Error bars, standard deviation ^∗^ *P* < 0.05, ^∗∗^ *P* < 0.01.

In CM of IFN*α*-AF-MSCs from P1-P5, constant IFN*α* expression was monitored by ELISA test. Levels of expressing IFN*α* were stable from P1-P5 IFN*α*-AF-MSCs (11490.5 ± 107.5 to 11,422 ± 68.5 pg/ml, *P* > 0.05, Figure 3(a)) while hardly any IFN*α* expressed was tested in CM of AF-MSCs (911.5 pg/ml, *P* < 0.01, Figure 3(a)).

The growth curves of both AF-MSCs and IFN*α*-AF-MSCs were roughly shaped “S” (Figure 3(b)), and no significant decrease in MSC proliferation was observed in the presence of IFN*α* production. The cells stayed in detention period after passaging for 2 days, then turned to logarithmic phase, and no longer increased seven days later. The proliferation of cells in the tenth generation was slower than in the fifth generation.

To assess the potential therapeutic effect of IFN*α*-AF-MSCs on the inhibition of tumor cell proliferation, the IFN*α*-overexpressing AF-MSCs were cocultured with HeLa cells in a Transwell system, and the apoptotic population of the tumor cells was detected by flow cytometry via annexin/propidium iodide (PI) staining. The results presented in Figure 3(c) indicated that the coculture with IFN*α*-AF-MSCs caused a large increase in early (annexin V+/PI−) and late (annexin V+/PI+) apoptoses in HeLa cells. The total percentage of apoptotic (early and late apoptoses) HeLa cells increased from 3.45 ± 1.44% to 84.57 ± 1.67% in the presence of IFN*α*-AF-MSCs (*P* < 0.01) (Figure 3(c)). In contrast, the coculture of HeLa cells with AF-MSCs induced much lower apoptosis in the cervical cancer cells (8.12 ± 0.73%), and this apoptotic rate was still significantly higher than that observed in HeLa cells that were cultured alone (*P* < 0.05).

### 3.5. Engraftment of IFN*α*-AF-MSCs Significantly Reduced the Size of HeLa Xenografts in Mice

To explore the efficacy of the cell-based therapy of IFN*α*-AF-MSCs, AF-MSCs (*n* = 12) or IFN*α*-AF-MSCs (*n* = 15) were administered intravenously (i.v.) into the tail veins of tumor-bearing mice. Tumor-bearing mice that received no MSCs were used as controls. An i.v. injection of 1 × 10^6^ MSCs was administered weekly for 3 rounds, and the mice (*n* = 3 in the control group, *n* = 5 in other two groups) were sacrificed 7 days after the last injection of MSCs for tumor collection and tumor apoptosis analysis. Tumor size was measured throughout the experimental period.

**Figure 4:**
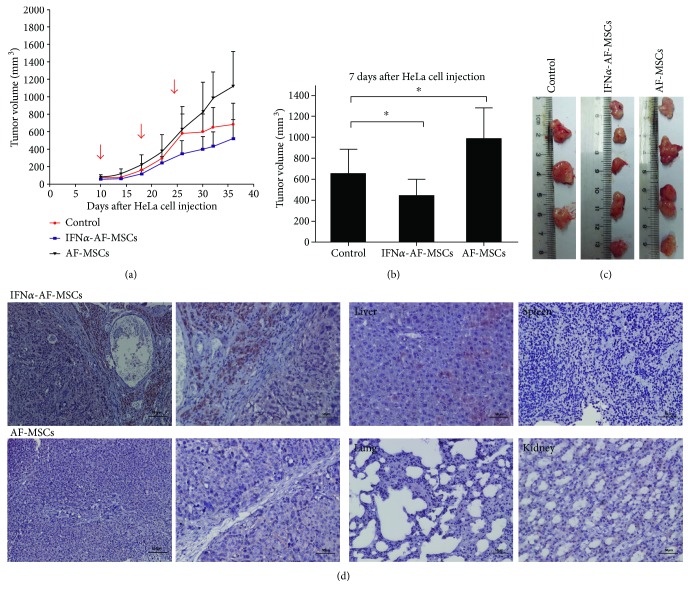
Effects of IFN*α*-AF-MSCs and AF-MSCs on tumor growth in a mouse model. (a) Comparison of the growth of tumors engrafted with different MSCs for up to 36 days after the inoculation of HeLa cells. The tumors were measured based on their size under the skin. The MSC injection time points are indicated by arrows. Error bars, standard deviation. (b, c) Comparison of the tumor volume from mice sacrificed on day 32 (7 days after the last injection of MSCs). Error bars, standard deviation. HeLa cells (1 × 10^7^/ml) were injected subcutaneously into nude mice (day 0). After 10 days, when the tumors were palpable, 1 × 10^6^/ml AF-MSCs (*n* = 12) or IFN*α*-AF-MSCs (*n* = 15) were administered i.v. into the tail vein of the tumor-bearing mice every seven days. This procedure was performed three times. Ten mice that were not administered with MSCs were designated as the control group. Some mice (*n* = 3 in the control group and *n* = 5 in the other three groups) were sacrificed seven days after the last injection of stem cells for tumor collection. (d) Secreted IFN*α* was detected by immunohistochemistry in tumor tissue in mice receiving IFN*α*-AF-MSCs (*n* = 5), and very little IFN*α* was detected in the liver, none in the spleen, kidney, or lung. No IFN*α* was found in the mice of AF-MSCs group (*n* = 5). Tissues were collected on day 7 after the last injection of IFN*α*-AF-MSCs (*n* = 5) or AF-MSCs (*n* = 5).

As shown in Figure 4, before the administration of MSCs, there was no significant difference in the size of tumors obtained from the mice from the four groups. After 3 injections of MSCs separated by intervals of 7 days, the tumor sizes of the mice that received IFN*α*-AF-MSCs started to show significant difference compared with those of the control group (*P* < 0.05) since day 20. The tumor sizes were measured on day 32 (7 days after the last injection) as shown in Figure 4(b), and the tumors from the IFN*α*-AF-MSC group (*n* = 15) were 434.99 ± 165.79 mm^3^ significantly smaller than those from the untreated group (653.89 ± 227.84 mm^3^) (*P* < 0.05). In addition, the difference in tumor size between the mice treated with IFN*α*-AF-MSCs and the untreated mice was significant when taken totally through the observation period of 52 days (Figure 4(a)).

### 3.6. AF-MSCs Enhanced the Size of HeLa-Derived Tumors in Mice

In contrast to the inhibitory effect of IFN*α*-AF-MSCs on the growth of HeLa xenografts in mice, the untransfected AF-MSCs significantly improved tumor growth. For the whole observation, the average tumor size in the AF-MSC group (364.72 ± 272.51) was significantly greater than that in the IFN*α*-AF-MSC (260.93 ± 189.12, *P* < 0.05). In particular, when the growth rate of tumors in the control group decreased beginning on day 26, the tumors in the AF-MSC-injected mice continued increasing in size and became significantly larger than those in the control mice.

### 3.7. Secreted IFN*α* Was Concentrated in Tumor Sites in IFN*α*-AF-MSC-Treated Model Mice

To confirm that the therapeutic effect of IFN*α*-AF-MSCs was the result of IFN*α* concentration in local tumor, secreted IFN*α* was detected by immunohistochemistry in tumor tissue, liver, spleen, kidney, and lung of tumor-bearing mice. Tissues were collected on day 7 after the last injection of IFN*α*-AF-MSCs (*n* = 5) or AF-MSCs (*n* = 5). In mice receiving IFN*α*-AF-MSCs, a large amount of secreted IFN*α* were found in tumor sites, especially in perivessel and stromal region (Figure 4(d)). A little IFN*α* was detected in the liver, and no IFN*α* was found in the spleen, kidney, or lung (Figure 4(d)). What is more, no IFN*α* was found in mice receiving AF-MSCs, neither in tumors, nor in organs (Figure 4(d)).

### 3.8. IFN*α*-AF-MSCs and AF-MSCs Enhanced Cell Apoptosis of Tumors in Model Mice

To explore the mechanism of the inhibitory effect of IFN*α*-AF-MSCs on the tumor growth of HeLa xenografts, the TUNEL assay for apoptotic cells and IHC for oncogenic proteins (c-Myc, p53, and Bcl-2) were conducted. Tumors from mice receiving IFN*α*-AF-MSCs (*n* = 5) or AF-MSCs (*n* = 5) were removed on day 7 after the last injection of MSCs, and paraffin sections were used for the in situ TUNEL assay and IHC. Tumors from the tumor-bearing mice that received no MSCs (*n* = 3) were analyzed as controls.

**Figure 5:**
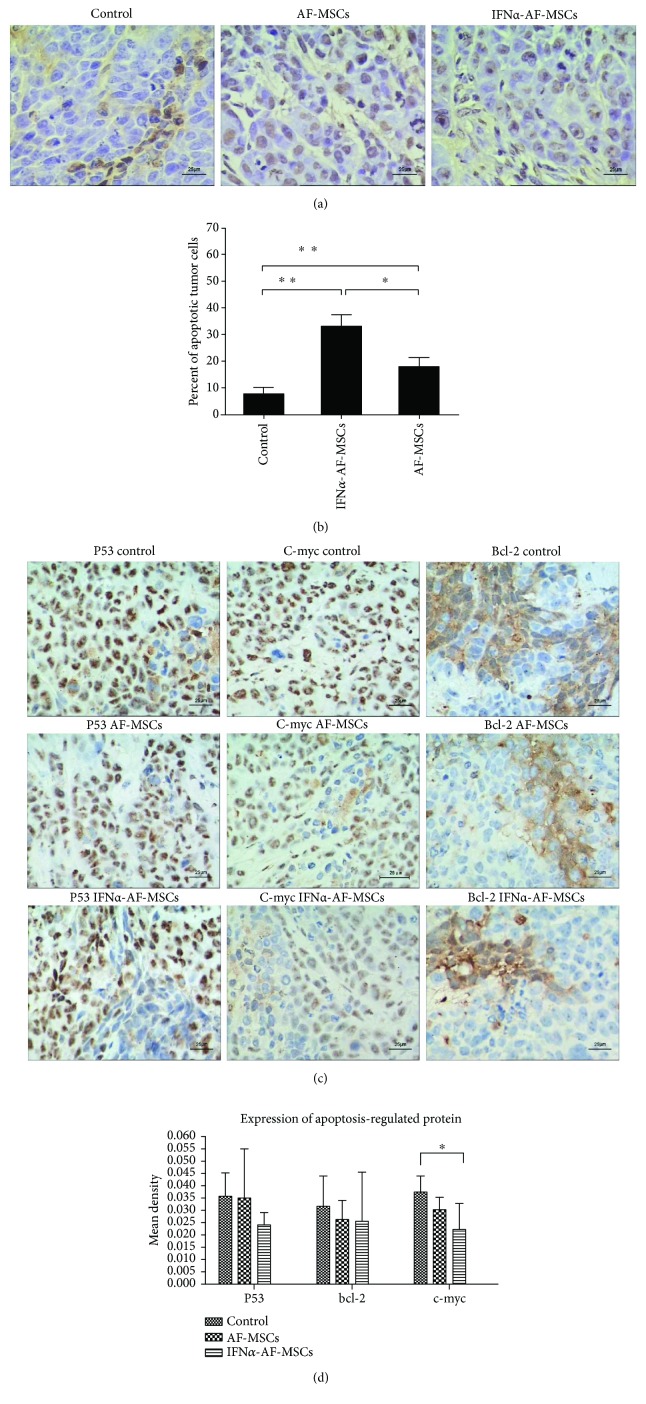
Increased cell apoptosis and reduced c-Myc expression in tumors from mice engrafted with IFN*α*-AF-MSCs. (a) TUNEL assay of tumors harvested on day 7 after the last i.v. administration of MSCs. Bars, 25 *μ*m. (b) Comparison of apoptotic cells in tumors taken from each group of mice. For each tumor, TUNEL-positive cells were counted in five randomly selected areas at ×400 magnification. Error bars, standard deviation. (c) The expression of c-Myc, p53, and Bcl-2 in the tumors. The solid tumors that had developed from 1 × 10^7^/ml HeLa cells administered subcutaneously to nude mice were removed and processed into paraffin sections for further analysis. The tumors were collected one week following the three weekly injections of 1 × 10^6^ AF-MSCs or IFN*α*-AF-MSCs (*n* = 5 per group). Tumors from three untreated mice were analyzed as controls. Tumor sections that were prepared in parallel were used for IHC using the corresponding specific antibodies and isotype control antibodies. Bar, 25 *μ*m. (d) Quantified results of c-Myc, p53, and Bcl-2 expression presented as the mean density. The stained sections were read by a microscope and quantified using the Image-Pro Plus (IPP) 6.0 software. The mean densities of 10 randomly selected fields at ×400 magnification in each tumor. All harvested tumors were analyzed. Error bars, standard deviation ^∗^ *P* < 0.05, ^∗∗^ *P* < 0.01.

As shown in Figures 5(a) and 5(b), the engraftment of IFN*α*-AF-MSCs induced a significantly higher proportion of apoptotic cells (32.76 ± 4.67%) in tumors than that in tumors of untreated mice (7.51 ± 2.67%, *P* < 0.01) and MSC-treated mice (17.63 ± 3.69%, *P* < 0.05). Interestingly, although the administration of AF-MSCs largely enhanced tumor growth, they also induce high level of apoptosis (17.63 ± 3.69%) in the tumor cells. Meanwhile, the effect of MSCs or IFN*α*-overexpressing MSCs on the expression levels of three proteins related to ontogenesis (c-Myc, p53, and Bcl-2) was examined by IHC. The expression levels of these proteins were analyzed by quantifying the density of positively stained cells using the Image-Pro Plus (IPP) 6.0 software. The results demonstrated that the mean density of c-Myc in the tumor sections prepared from the IFN*α*-AF-MSC-engrafted mice (0.0223 ± 0.0101, *n* = 5) was significantly lower than that of the control group (0.0374 ± 0.0064, *n* = 5, *P* < 0.05), but no significant differences were observed between either of the other 2 groups. There were no significant differences in the expression levels of p53 and Bcl-2 (Figures 5(d) and 5(e)).

### 3.9. IFN*α*-AF-MSCs Significantly Reduced Angiogenesis in the Tumors of Model Mice

**Figure 6:**
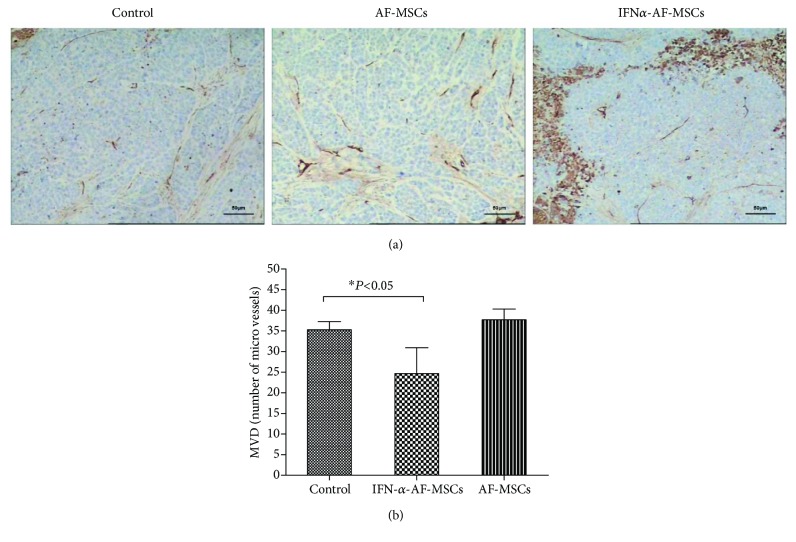
Reduction of tumor angiogenesis by IFN*α*-AF-MSCs in a mouse model. (a) Representative images of areas with the highest density of microvessels in each group. Bar, 25 *μ*m. (b) Comparison of MVD between the different experimental groups. Error bars, standard deviation. Solid tumors in nude mice formed by 1 × 10^7^/ml of subcutaneously administered HeLa cells were removed and processed into paraffin sections to measure microvessel density (MVD). The tumors were collected one week following the three weekly injections of 1 × 10^6^ AF-MSCs and IFN*α*-AF-MSCs (*n* = 5 per group), and tumors from three untreated mice were analyzed as controls. Micro-blood vessels were indicated by IHC staining for CD34, and individual microvessels in at least five fields in each tumor were counted at ×200 magnification. Both isolated endothelial cells and luminal microvascular structures were considered countable vessels. ^∗^ *p* < 0.05.

To investigate whether the restriction of angiogenesis is associated with the inhibition of tumor growth by IFN*α*-AF-MSCs, the number of blood vessels in tumor sections from MSC-engrafted and ungrafted mice was examined. Microvessel density (MVD) was assessed for tumor angiogenesis analysis using the CD34 antibody. The paraffin sections used in this experiment were obtained from the same tumors that were used for the apoptosis analysis. The counting method introduced by Weidner et al. [26] was utilized. As shown in Figure 6, at ×200 magnification under light microscopy, there were significantly fewer countable blood vessels in the tumors of IFN*α*-AF-MSC-engrafted mice (25 ± 6) compared with those in the untreated mice (35 ± 2, *P* < 0.05). However, no significant difference in MVD was observed in AF-MSC-engrafted group (38 ± 3) compared with that in the untreated controls (*P* > 0.05).

### 3.10. Engraftment of AF-MSCs Did Not Result in Tumor Formation in Model Mice

To investigate whether the engrafted MSCs could form tumors or cause significant side effects in the recipients, 4-week-old nude mice were subcutaneously inoculated with 1 × 10^7^ AF-MSCs or IFN*α*-AF-MSCs (*n* = 4 for each group). Small, hard swelling resembling nonspecific inflammation was observed in the first 3 days after transplantation, but this gradually disappeared. The mice were kept for 50 days before being sacrificed. Neither obvious weight loss nor other symptoms of poor health were observed during the experimental period. No visible solid tumors were found around the injection sites or in any other organs in any of the three groups of mice. The pathological diagnosis via hematoxylin-eosin- (HE-) stained slides confirmed the absence of tumor formation as well (Figure S2). These results suggest that both hAF-MSCs and those engineered to express IFN*α* are potentially safe for use in transplantation therapy.

## 4. Discussion

Although IFN*α* is effective in treating cervical intraepithelial neoplasia (CIN) and cervical cancer in clinical trials [1, 29], its cytotoxic side effects, which result from high doses of administration, are inevitable. Thus, cell-based-targeted treatment was explored. Due to their innate capacity for tumor tropism, MSCs derived from various sources, such as the BM and umbilical cord, have been widely studied as delivery vehicles for therapeutic agents in the treatment of various types of cancers [11, 12, 16, 30]. Because AF-MSCs are easily obtained and have stable self-renewal and proliferative properties, they have great potential in treatment application. Our study showed that modified AF-MSCs expressing IFN*α* were effective in suppressing cervical tumor growth.

Since cervical cancer is mostly caused by HPV infection, we chose HeLa cell line, which was originated from human papillomavirus 18-affected cervical cells, as our research subject. Our study demonstrated that AF-MSCs selectively migrated to the tumor site after engraftment and that this tropism capacity was retained even after the MSCs were genetically engineered to express IFN*α*. In addition, we found that AF-MSCs and IFN*α*-AF-MSCs were specifically detected in the tumor periphery, inside stromal regions, and around areas of necrosis. This finding is in agreement with many previous studies on the homing capacity of MSCs [11–13]. The MSCs that engrafted at the tumor site were believed to contribute to the population of stromal fibroblasts and participate in tumor formation [3]. The solid tumor environment is composed of malignant cells and matrix components, and MSCs have the potential to transform into activated myofibroblasts or differentiate into fibrocytes, which produce ECM components, and perivascular or vascular structures. The tumor microenvironment is a site where chemokines and cytokines, such as VEGF, urokinase plasminogen activator (uPA), IL-8, transforming growth factor beta-1 (TGF-b1), and monocyte chemotactic protein-1, are abundantly secreted, and these molecules may induce the homing of MSCs [13, 31].

Because MSCs are capable of selectively engrafting and participating in tumor stroma development, this recruitment was employed in a “Trojan horse” approach in cell-based-targeted therapy. Our study demonstrated that AF-MSCs engineered to express IFN*α* were effective in tumor suppression in a mouse model of cervical cancer. When HeLa cells were cocultured with IFN*α*-AF-MSCs, the large amount of IFN*α* produced by IFN*α*-AF-MSCs induced high levels of tumor cell apoptosis. In IFN*α*-AF-MSC-treated mice, secreted IFN*α* was only found assembled in tumor sites and even presented similar distribution as AF-MSCs showed in the previous tropism investigation. Meanwhile, significant decrease in tumor size was also observed in the IFN*α*-AF-MSC group. Overall, IFN*α*-AF-MSCs' significant therapeutic effect on tumor suppression confirmed that the targeted delivery of IFN*α* by IFN*α*-AF-MSCs was efficient, even in the complex in vivo environment consisting of metabolic and cytokine interactions. What is more, we also found larger areas of necrosis inside the tumor mass in IFN-MSC-treated mice than in other groups which implies that the efficacy of IFN-MSC treatment was not simply displayed through or judged on the visual appearance of the tumor mass.

The tumor suppressive effects of IFN*α*-overexpressing MSCs could be due to an induction of damage to the tumor cells (such as higher apoptosis, as demonstrated in both in vitro and in vivo experiments) and/or inhibition of the proliferation of the tumor cells (such as a reduction in the supply of nutrition and hypoxia environment). Tumor suppressor gene p53 and apoptosis control gene bcl-2 are two important genes for cell survival. They were reported to be involved in cervical cancer carcinogenesis [32]. P53 expression was detected in early stages in cervical cancer carcinogenesis and has a role in progression to cervical cancer [32]. Protooncogenes such as c-Myc direct changes in metabolism and protein synthesis supporting enhanced proliferation rates. The gene copy number gain of c-Myc was significantly higher in the cervical lesion of advanced histologic grade [33, 34]. In our research, c-Myc expression dropped in cervical cancer cells as a result of IFN*α*-overexpressing MSC treatment, while neither p53 nor bcl-2 is related. It is also known that enhanced angiogenesis is essential for tumor growth. Previous study evaluating the potential of bone marrow-derived mesenchymal stem cells (MSC), genetically modified to express interferon (IFN)-alpha, for the treatment of lung metastasis in an immunocompetent mouse model of metastatic melanoma, indicated that the antitumor effect might be related to a potential decrease of angiogenesis and/or induction of tumor cell apoptosis [35]. Therefore, we evaluated the effect of IFN*α*-AF-MSCs on angiogenesis in our xenograft model by examining the MVD in the tumor and found a significant reduction in blood vessels compared with that in the control groups. This reduced angiogenesis could, in turn, promote the apoptosis of tumor cells induced by the IFN*α*-AF-MSCs. Our finding of angiogenesis inhibition was in line with a previous study [13] that used a bladder tumor model, which demonstrated that IFN*β*-AF-MSC treatment resulted in a reduction in vascularization.

The experimental results regarding whether MSCs alone promote or inhibit tumor growth have been controversial. MSCs were shown to suppress tumor growth in some models of tumors, such as glioma, Kaposi's sarcoma, malignant melanoma, Lewis lung carcinoma, and colon carcinoma [15, 30, 36]. These studies revealed that the mechanisms for suppression of tumor growth varied and included inhibition of certain enzymatic proteins and concentration-dependent inhibition of angiogenesis. However, a few studies have reported that MSCs can contribute to tumor growth [37–39]. Lis et al. demonstrated that the properties of ovarian cancer cells, namely, metastatic abilities (adhesion, migration, and invasion), proliferation, and chemoresistance, can be promoted by MSCs [37]. This promoting effect was considered to be due to the induction of cytokines, including vascular endothelial growth factor (VEGF) and basic fibroblast growth factor (bFGF), which induce the division and blood vessel formation of endothelial and stroma cells under abnormal conditions, such as wound healing or tumorigenesis [3]. In fact, the tumor type, model, route of MSC transplantation, cell dose, and time course all tended to influence the experimental outcomes [12]. Our study showed that the AF-MSCs significantly increased tumor size. However, interestingly, in situ TUNEL assay showed that AF-MSCs alone also showed induction of apoptosis. According to our previous MVD detection results, tumors of the AF-MSC group did not enjoy a higher standard of angiogenesis inside, even if they showed in the greatest size among all the groups. Thus, relative lack of blood and nutrition supply might occur and induce an increase in the percentage of apoptotic cells in solid tumors. Anyway, tumor-promoting effect of AF-MSCs was reversed after they were modified to express IFN*α*, and these stem cells demonstrated an inhibitory effect on tumor cell growth.

In addition, considering the potential tumorigenesis risk of AF-MSCs and gene-modified IFN*α*-AF-MSCs, we performed further experiments to evaluate the possibility that AF-MSCs may form tumors. Up to 1 × 10^7^ of AF-MSCs and IFN*α*-AF-MSCs were subcutaneously administered to nude mice, and no visible signs of tumorigenesis were identified at the injection sites during the 50-day observation period; pathological diagnosis further verified that no tumorigenesis existed, either in the injected subcutaneous tissue or in the collected organs. Therefore, we deduced that even if AF-MSCs promoted tumor growth in vivo, they do not contribute to tumorigenesis in regions of nonmalignant cells. Collectively, we assume that IFN*α*-AF-MSCs are safe and effective in restricting cervical cancer cell growth in short terms.

In conclusion, our study demonstrated that IFN*α*-AF-MSCs are capable of homing to the tumor site in a HeLa cell xenograft model and exhibit therapeutic efficacy in tumor suppression. We also demonstrated that although AF-MSCs alone can selectively engraft into the tumor site and participate in tumor construction, they are safe and effective after being genetically engineered to express IFN*α*, with the aim of treating cervical cancer. Our data suggest that the tumor suppression efficacy of IFN*α*-AF-MSCs is attributed to angiogenesis reduction, as well as the induction of apoptosis and inhibition of cell proliferation in tumor cells mediated by these IFN*α*-overexpressing stem cells. Further investigation is needed to determine the optimal cell dose and administration routes, as well as how to prolong the duration of therapeutic efficacy. Additionally, the long-term risk of further immunosuppression triggered by AF-MSCs should also be taken into consideration when AF-MSCs are administered to patients with immunodeficiency [18]. Ultimately, further studies utilizing other therapeutic agents in different tumor models are necessary to validate the efficacy of AF-MSC-based-targeted therapy.

## Supplementary Materials

Supplementary Materials Figure S1: identification of AF-MSCs. (a) Identification of specific cell surface markers for AF-MSCs by flow cytometry. AF-MSCs of the 1st, 3rd, 7th, and 15th generations were stained for CD90, CD105, CD73, HLA-ABC, CD34, CD14, CD45, and HLA-DR and were analyzed by flow cytometry. AF-MSCs were CD90^+^, CD105^+^, CD73^+^, HLA-ABC^+^, CD34^−^, CD14^−^, CD45^−^, and HLA-DR^−^. IFN*α*-AF-MSCs are consistent with the MSC null ones in terms of stem cell markers, which proved the cell stability. Representative results from one of three comparable experiments are shown. (b) Osteogenic differentiation of AF-MSCs. AF-MSCs were stained with Alizarin red S after being cultured in osteogenic medium for 21 days. Osteogenic induction was shown by positive staining (indicated by arrows), which occurs as a result of mineralized differentiation. The photograph is a representative image of two independent experiments. Figure S2: absence of tumor formation from inoculated AF-MSCs. H&E staining of tissues from mice that received subcutaneous injection of 1 × 107 AF-MSCs or IFN*α*-AF-MSCs. Samples were obtained from four mice 50 days after injection. The images show representative fields of the tissues from the injected sites, specifically the liver, kidney, spleen, and lung. Bar: 50 *μ*m.
