# Supplementary figures and images for: IFNα-Expressing Amniotic Fluid-Derived Mesenchymal Stem Cells Migrate to and Suppress HeLa Cell-Derived Tumors in a Mouse Model
Source: Stem Cells Int. 2018 Apr 2;2018:1241323. doi: 10.1155/2018/1241323 (PMC5901954; doi:10.1155/2018/1241323)

Fig S1


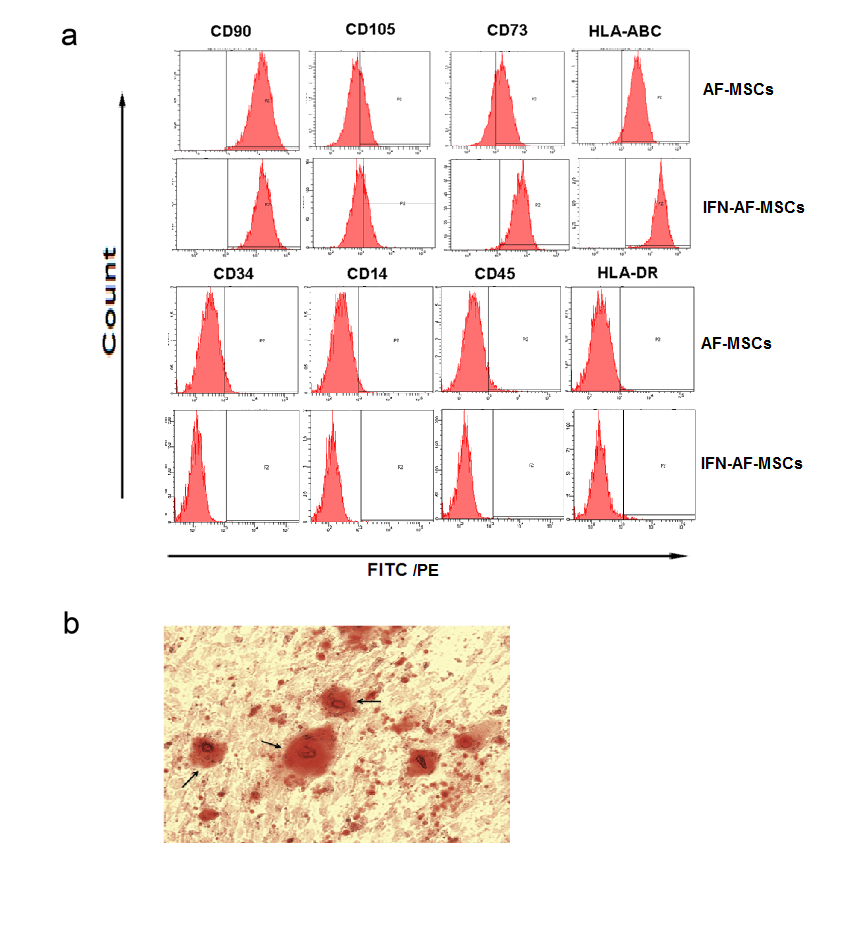


Fig S2


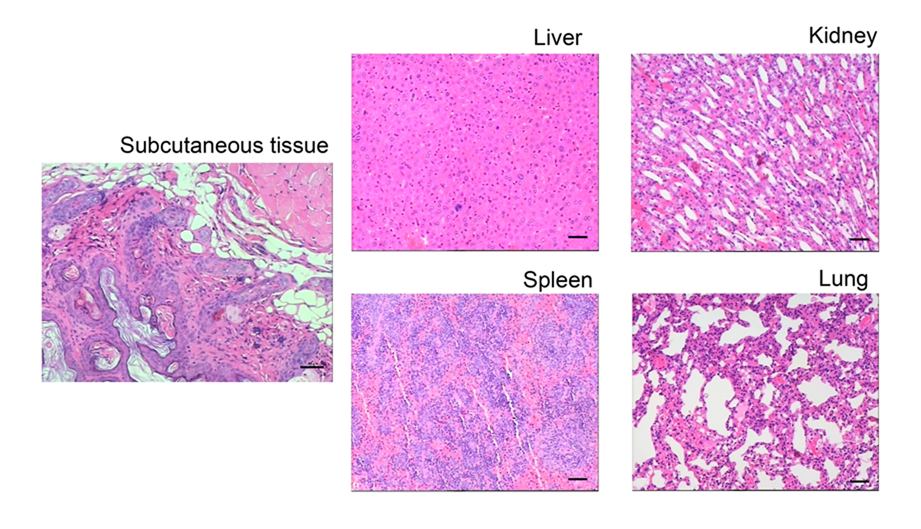


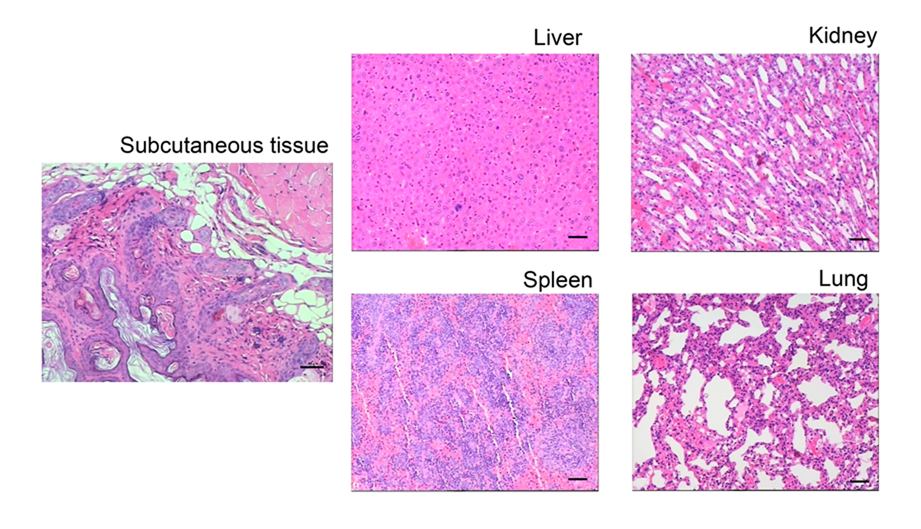
Fig S2

Supplement: Supplementary Materials — Figure S1: identification of AF-MSCs. (a) Identification of specific cell surface markers for AF-MSCs by flow cytometry. AF-MSCs of the 1st, 3rd, 7th, and 15th generations were stained for CD90, CD105, CD73, HLA-ABC, CD34, CD14, CD45, and HLA-DR and were analyzed by flow cytometry. AF-MSCs were CD90+, CD105+, CD73+, HLA-ABC+, CD34−, CD14−, CD45−, and HLA-DR−. IFNα-AF-MSCs are consistent with the MSC null ones in terms of stem cell markers, which proved the cell stability. Representative results from one of three comparable experiments are shown. (b) Osteogenic differentiation of AF-MSCs. AF-MSCs were stained with Alizarin red S after being cultured in osteogenic medium for 21 days. Osteogenic induction was shown by positive staining (indicated by arrows), which occurs as a result of mineralized differentiation. The photograph is a representative image of two independent experiments. Figure S2: absence of tumor formation from inoculated AF-MSCs. H&E staining of tissues from mice that received subcutaneous injection of 1 × 10^7^ AF-MSCs or IFNα-AF-MSCs. Samples were obtained from four mice 50 days after injection. The images show representative fields of the tissues from the injected sites, specifically the liver, kidney, spleen, and lung. Bar: 50 μm. [file 1241323.f1.docx]
